# On the Nature and Structural Characteristics of Cancer, and of Those Morbid Growths Which May Be Confounded with It

**Published:** 1840-07

**Authors:** 


### Art. XVI.

*On the Nature and Structural Characteristics of Cancer, and of those Morbid Growths which may be confounded with it.* By J. Müller, m.d. Translated from the German, with Notes, by Charles West, m.d. Graduate in Medicine of the University of Berlin. Part I.—*London*, 1840. 8vo, pp. 182.

The design which the well-known author of this work had in view was to discover, by microscopic and chemical examination some more certain characters than are at present known for the diagnosis of those morbid growths which are often nearly similar in their evident structure though widely different in their pathological relations. In this design, however, his success appears to us to be very limited. In this first part of the work we cannot find that any sign is yet discovered in the microscopic or chemical characters of tumours which will suffice for a strict decision in the only cases in which, to a practised eye, any difficulty has hitherto existed. The subjects here considered are the various forms of cancer (including all malignant growths); and the distinctions between them and cartilaginous, fatty, and cysto-sarcomatous tumours. But we believe that few persons, in the habit of examining tumours by the means commonly employed, would have much difficulty in forming as correct a diagnosis between cancer and any of these growths as the author by his more minute investigations pretends to establish. And in regard to some of the tumours which are to be considered in a future part of the work, the author, in speaking of the osteo-sarcomatous growths which occur especially about the bones of the face, confesses, as less accurate observers do, that "it is far from an easy matter to distinguish between these growths and those tumours of the bones which are really of a cancerous nature," (p. 137;) and in other parts speaks not unfrequently of similar difficulties. Between many tumours, therefore, the differences, important as they must be, seem to lie still deeper than microscopic sight or proximate analysis have yet reached.

But if this be the case with growths removed from the body, still less does the present work add to the means of determining the nature of tumours before extirpation, when, more than at any other time, an exact diagnosis is desirable. The author justly says that "microscopical and chemical analysis can never become a means of surgical diagnosis; it were ridiculous to desire it or to suppose it practicable." (p. 5.) At the same time it is to be remembered that in nearly all objects of natural history differences of minute structure are accompanied by almost as constant diversities of general external appearance. To take an example, to which he himself, for a different illustration, alludes,—the botanist, to determine the species of a minute moss or conferva, does not need to subject every specimen that he meets with to a microscopic examination, but he knows that each species has, with a certain minute structure, a peculiar general aspect, by which he can at once and at first sight determine its name; and he reserves the microscope only for a last resource in case of unusual doubt. And so should it be in the diagnosis of tumours; but in the determination of these general characters the present work rarely goes a step beyond those that are already well known.

However, in pointing out these deficiencies of the work, which will scarcely detract from its value in the opinion of the pure pathologist, we are far from wishing it to be thought that Professor Müller has not made another important contribution to science. Independently of the accurate description of the structure and mode of growth of many varieties of tumours, and of the more solid foundation which he has laid for a good classification of them all, some of the generalizations at which he has arrived are novel and of considerable value. The chief of them relates to the elementary constitution and laws of development of all the tumours he has yet examined, and which are proved to be in every apparent circumstance similar to those which prevail in the normal tissues. He has determined, with evident truth, "that no division of pathological structures into homologous and heterologous can be established. The most innocent growths do not differ in their minute elements, nor in their origin, from carcinoma." (p. 24.) Indeed the elementary structures of all the morbid growths hitherto examined resemble in every respect the structures presented in the several stages of development of the elements of the healthy tissues of the body. "Nuclei, cells, caudate bodies, fibres, and crystals are the only bodies which have hitherto been discovered in morbid growths, (p. 9); and many of them present some of the most remarkable illustrations of the universal law of development from cells, of which we gave in our last number a full account from the works of Schwann and Schleiden, and of which, we may observe, some knowledge is essential to a just appreciation of the greater part of Prof. Müller's investigations.

All tumours then are developed from the same element (nucleated cells), and according to the same laws as the healthy tissues. Cells growing upon nuclei, and developing new cells within themselves, or elongated into caudate or spindle-shaped bodies, or in a still higher stage of development forming fibres, constitute the main structure of all morbid growths. Blood-vessels, which have been by many regarded as having the initiative in these diseases, are late formations, being produced in the masses of elementary structures, just as they are in those of the embryo, subsequently to many and important changes which are effected without their aid.

In general, however, the development of the elementary structure of morbid growths is checked at an early stage. The cell is by far the most frequent element of morbid growths, and sometimes cells alone compose their tissue, as in the laminated fatty tumour (cholesteatoma), the cellular sarcoma, the variety of cancer which the author terms carcinoma alveolare, and some others. In these instances cells cohering by their walls form the important parts of the structure, while fibres of cellular tissue serve only to form membranes uniting together its several lobules. In other cases cells elongated into caudate bodies are the chief element, as in fungus medullaris and sometimes in melanosis, and in many non-carcinomatous growths. In other examples again the cells are fully developed into fibres, as in the fibrous or desmoid tumour, the carcinoma fasciculatum, &c.

Nor is the fundamental similarity between morbid growths and the normal tissues confined to the structure of their elements; there is no essential difference in their chemical composition. All tumours, whether innocent or malignant, may be divided according to their main composition, as healthy tissues may, into fatty, gelatinous, and albuminous; and in none is there any peculiar proximate principle discoverable forming a considerable portion of its composition.

But, however these facts may appear to simplify the history of morbid growths, it must be confessed that they effectually destroy for the present all hope of being able by minute observations to distinguish the broad differences of *innocent* and *malignant*, which constitute their most important character, not only as subjects of surgical practice but also as objects of pathological science. And hence it is that Müller's definitions of cancer are based on no other characters than those which have been long known and appreciated; viz. its tendency to the destruction of all the tissues around it, and its liability to return after extirpation. Neither in the general observations, by which the description of the six varieties which he makes is prefaced (p. 28), nor in the summary which at its conclusion he gives on the nature of carcinoma (p. 78), is any attempt made to found a distinction between cancer and other morbid growths upon any of the minute observations to which the author has devoted himself.

The part of the work in which fewer deficiencies are evident is that which relates to innocent growths, including enchondroma, or cartilaginous tumours, lipoma or fatty tumours, and cysto-sarcoma. Of all these, and especially of the first, the description is considerably more minute and clear than any that we have yet met with, and is sufficient to establish satisfactorily their essential nature, and the history of their development.

With reference to the translation we are bound to say that Dr. West has acquitted himself very creditably. He has rendered some of the harshest and most crabbed German we ever read into smooth and pure English; and the few notes that he has added are all judicious and useful. Dr. West was equally successful in his translation of Naegele's excellent work on Obstetric Auscultation.

---

